# Parallel flowering time clines in native and introduced ragweed populations are likely due to adaptation

**DOI:** 10.1002/ece3.6163

**Published:** 2020-04-29

**Authors:** Brechann V. McGoey, Kathryn A. Hodgins, John R. Stinchcombe

**Affiliations:** ^1^ Department of Ecology and Evolutionary Biology University of Toronto Toronto ON Canada; ^2^ School of Biological Sciences Monash University Clayton VIC Australia; ^3^ Koffler Scientific Reserve University of Toronto Toronto ON Canada

**Keywords:** adaptation, clines, GBS, genotyping‐bysequencing, invasive species, population differentiation, ragweed

## Abstract

As introduced species expand their ranges, they often encounter differences in climate which are often correlated with geography. For introduced species, encountering a geographically variable climate sometimes leads to the re‐establishment of clines seen in the native range. However, clines can also be caused by neutral processes, and so it is important to gather additional evidence that population differentiation is the result of selection as opposed to nonadaptive processes. Here, we examine phenotypic and genetic differences in ragweed from the native (North America) and introduced (European) ranges. We used a common garden to assess phenotypic differentiation in size and flowering time in ragweed populations. We found significant parallel clines in flowering time in both North America and Europe. Height and branch number had significant clines in North America, and, while not statistically significant, the patterns in Europe were the same. We used SNP data to assess population structure in both ranges and to compare phenotypic differentiation to neutral genetic variation. We failed to detect significant patterns of isolation by distance, geographic patterns in population structure, or correlations between the major axes of SNP variation and phenotypes or latitude of origin. We conclude that the North American clines in size and the parallel clines seen for flowering time are most likely the result of adaptation.

## Graphical abstract

Clines in invasive species can be due to adaptation or non‐adaptive mechanisms. We use a combination of common garden field experiments and population genetic analysis of GBS data to show that clines in common ragweed are likely adaptive.

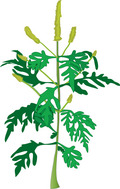

## 1 INTRODUCTION

Invasive species are both biological disasters and curiosities. In addition to the dual economic and ecological damage introduced plants can cause when they proliferate, they also offer opportunities to study evolutionary ecology during colonization (Callaway & Maron, 2006). For example, invasions provide ideal systems to study the effects of reproductive isolation, how dispersal affects species distributions, and the effect of a new individual species on an ecosystem (Sax et al., 2007). Evolutionary biologists have garnered insights from introduced species for decades by studying patterns of variation, interactions with native species, and comparing native and introduced populations (Huey, Gilchrist, & Hendry, 2005). Clines in introduced populations offer an opportunity to study parallel evolution, the rate and predictability of local adaptation, and whether phenotypic divergence is due to selection or stochastic, nonadaptive forces (Colautti & Lau, 2015; Huey et al., 2005; Samis et al., 2012). Here, we use a field common garden experiment and genotyping by sequencing (GBS) to investigate clines in introduced and native ragweed populations with the goal of distinguishing between adaptive and nonadaptive mechanisms underlying clinal variation.

Incorporating an evolutionary perspective into invasion biology is critical to understanding the course an invasion has taken and how it might continue to unfold. For example, the capacity of an introduced population to adapt to its new climate is important for its capacity to persist and spread (Colautti & Barrett, 2013). Adaptation to climate variables often leads to geographic differentiation, as climate and geography are strongly correlated (Endler, 1977). The common pattern of a gradient in traits or alleles over a geographic range (Huxley, 1938) is often interpreted as evidence of adaptive differentiation. Clines can be found in both Mendelian and quantitative traits, and there are hundreds of examples across a wide variety of taxa (Campitelli, 2013). Clines in introduced species, especially those that mirror geographic variation in the native range, are often perceived as evidence that introduced populations have adapted to their new environments (Colautti & Barrett, 2013; Samis et al., 2012). However, processes other than adaptation can also be responsible for both phenotypic and genetic clines, and need to be controlled for (Keller & Taylor, 2008). For example, phenotypic clines observed *in situ* could be caused by plastic responses to environmental variables, especially in plant species (Huxley, 1938), and neutral processes present through colonization could also produce clines (Keller, Sowell, Neiman, Wolfe, & Taylor, 2009; Santangelo, Johnson, & Ness, 2018; Vasemägi, 2006).

Distinguishing between the possible forces underlying clines can be achieved in several ways. Plasticity can be excluded by using a common garden to ensure that any differences between populations have a genetic basis (Lucek, Sivasundar, & Seehausen, 2014). Linking clines with natural selection and demonstrating a correspondence between the direction of selection and variation in phenotypes can also strengthen the case that a cline is the result of adaptation (Etterson, Delf, Craig, Ando, & Ohgushi, 2008). Parallel clines can also be interpreted as evidence of adaptation (Samis et al., 2012). Neutral markers can be used to rule out drift and support hypotheses that differences are due to adaptation (Campitelli & Stinchcombe, 2013; Keller & Taylor, 2008; Kooyers & Olsen, 2012; Le Gros et al., 2016; Lima et al., 2012). Likewise, Q_ST_‐F_ST_ comparisons can be used to compare molecular and quantitative genetic variation (Whitlock, 2008). Whereas F_ST_ examines differentiation at neutral markers, Q_ST_ is analogous but quantifies population divergence for quantitative traits (Spitze, 1993). Meta‐analyses have found that genetic differentiation among introduced populations is common and on average does not differ much in magnitude from divergence between native populations (Colautti & Lau, 2015). By using these methods, researchers have bolstered the argument that rapid adaptation occurs in non‐native species from plants to fruit flies (Colautti & Lau, 2015; Huey, Gilchrist, Carlson, Berrigan, & Serra, 2000; Montague, Barrett, & Eckert, 2008; Samis et al., 2012).

*Ambrosia artemisiifolia* (ragweed) is a globally invasive species with a wide range in its native continent of North America and a presence in Europe, Asia, and Australia (Friedman & Barrett, 2008). Past work on *A. artemisiifolia* has demonstrated parallel clinal patterns in flowering time in the native and European ranges (Hodgins & Rieseberg, 2011), and native and introduced Chinese ranges (Li, Zhang, & Liao, 2015). In this experiment, we examine variation in several quantitative traits across geography. To determine how quantitative variation may have been impacted by neutral processes, we use single neutral polymorphism (SNP) data to assess neutral genetic variation. We ask the questions: **Are there clinal patterns in quantitative traits, in the native and introduced European ranges? Are the patterns consistent with a history of selection in the introduced range, or nonadaptive processes?** Our results corroborate past results (Hodgins & Rieseberg, 2011; van Boheemen et al. 2018) of clinal variation in ragweed are likely due to adaptive differentiation rather than stochastic processes using independent experiments, analytical approaches, and biological samples (i.e., unique material and collections).

## 2 METHODS

### 2.1 Study species

*Ambrosia artemisiifolia* (common ragweed) is an annual outcrosser in the Asteraceae family (Bassett & Crompton, 1975; Friedman & Barrett, 2008). Ragweed is thought to have originated in the plains of North America and then spread eastward (Bassett & Crompton, 1975). In the modern era, ragweed has been accidently introduced to Europe, Asia, and Australia (MacKay & Kotanen, 2008). In Europe, ragweed has been present since at least the mid‐1800s, but propagule pressure (a composite measure of the individuals or seeds released in an introduction and the number of introductions (Lockwood, Cassey, & Blackburn, 2005)) increased dramatically in the mid‐20th century when ragweed seeds contaminated grains that were shipped from the Americas to Europe (Chauvel, Dessaint, Cardinal‐Legrand, & Bretagnolle, 2006). The geopolitical situation during that period meant that imports were coming from different areas into Western versus Eastern Europe. Two invasion centers resulted, with distinct genetic origins (Gladieux et al., 2011). In France, the epicenter of the invasion is the Rhône valley, where ragweed grows in very large populations along riverbanks (Chauvel et al., 2006; Thibaudon *et al.* 2013). In Eastern Europe, ragweed populations now extend north up into Poland and south into the Baltic states (Prank et al., 2013). In Hungary, the most impacted nation, it is the most widespread weed in surveys (Kiss & Béres, 2006) and over 80% of arable land is affected (Buttenschøn, Waldispuhl, & Bohren, 2010). Ragweed is thus one of the most problematic invaders in Europe: It causes allergies and is a significant agricultural weed.

### 2.2 Seed collection and preparation

**Figure 1:**
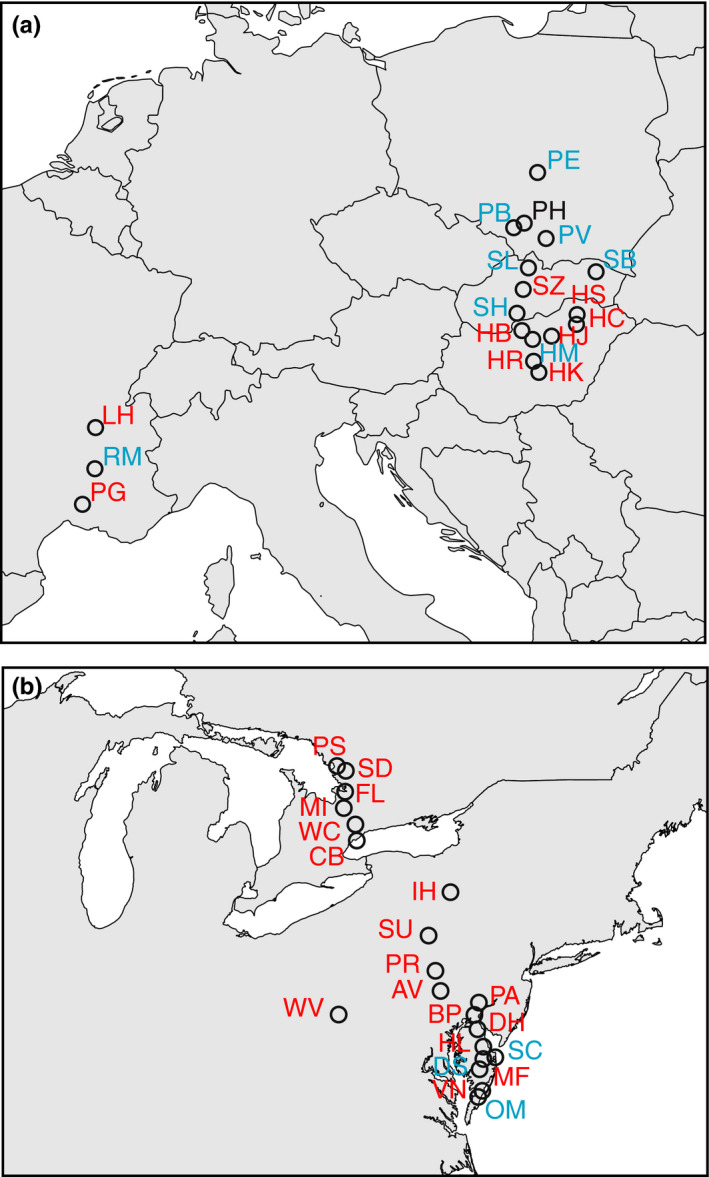
Map of population* Ambrosia artemisiifolia collection* sites from (a) native North American range and (b) invasive European range. Collection sites used only for the common garden are shown in blue. Sites used for both the common garden and SNP study are shown in red

In the autumns of 2012 and 2013, we collected seeds from a total of 20 native and 18 introduced populations (Figure1; population coordinates in Tables A1 and A2). In both ranges, the populations spanned ~ 7.5 degrees in latitude. These populations ranged from small (5 individuals) up to tens of thousands of individuals. When populations had fewer than twenty individuals, we collected seeds from all the plants. For larger populations, we collected from a random subset. Using methods adapted from Willemsen (1975) and Jannice Friedman (Queen's University, pers. comm.), we stratified seeds at 4°C for six months in plastic bags filled with silica and distilled water.

### 2.3 Common garden

For this experiment, we randomly chose 10 maternal families per population, except in cases where there were fewer than 10 families, in which case we used all available. We chose three germinants from each family (one for each of three blocks) for a total of 1,110 plants. Each individual was randomly assigned a position within a block. We planted germinants in seedling trays and kept in a greenhouse where we sprayed and bottom watered them for three weeks. At the end of June 2014, we planted seedlings into a plowed field at the Koffler Scientific Reserve (www.ksr.utoronto.ca; 44.803°N, 79.829°W). Blocks were subdivided into plots, each containing 64 plants in an 8x8 configuration, except for the final plot. We continued to remove interspecific vegetation and provide the seedlings with water for four weeks after transplantation to promote establishment.

### 2.4 Phenotypic traits

We measured final height, final number of branches, and date of first flower. Since the vast majority of ragweed plants are monoecious (Bassett & Crompton, 1975), we measured proxies of both male and female fitness. Male reproductive effort was estimated as the total inflorescence length, which is correlated with pollen production (Fumanal, Chauvel, & Bretagnolle, 2007). We estimated female reproductive output, using seed mass, which is highly correlated with seed number (*r* ^2^ = 0.96, *p* < .001) (MacDonald and Kotanen 2010). We ran initial models including latitude and block to assess whether block had a significant effect on traits; block was not significant for any trait and was thus dropped in subsequent models. To test for clines in phenotypic traits, we used linear regressions on maternal family means. For each continent, we conducted regressions for three phenotypic traits (height, flowering time, and branch number) and the two fitness traits on latitude. We used separate regressions for each continent rather than ANCOVA for three reasons: first, because the latitudinal ranges between Europe and North America were only partially overlapping; second, because the relationship between latitude and climate differs dramatically between Europe and North America; and third, because our goal was to test for clines independently in each continent. Similar results were obtained using population means for traits and latitude. Unless otherwise specified below, statistical analyses were conducted in R (R Development Core Team 2016).

### 2.5 Neutral markers

We collected leaf material from 180 ragweed plants from 26 populations (9 introduced, 17 native) grown in seedling trays in growth chambers at the University of Toronto. These plants were from a subset of the populations used in the common garden, but were separate plants. GBS library prep was conducted by the Hodgins lab at Monash University Australia. In brief, DNA was digested from the dried leaves and adapters were ligated to the strands. A double enzyme digest with Pst1 and Msp1 was implemented using the same protocol as in van Boheemen et al (2018). DNA libraries were sent to Genome Quebec for sequencing on an Illumina HiSeq 2,500, using PE125 sequencing.

We used Stacks (Catchen, Hohenlohe, Bassham, Amores, & Cresko, 2013) and Bowtie 2 (Langmead & Salzberg, 2012) to demultiplex, align to a reference genome provided by the Hodgins lab, and to calculate population genetic metrics. We checked the sequence quality using FastQC (Andrews, 2010) and samtools (Li et al., 2009). We converted between multiple formats using PGD Spider (Lischer & Excoffier, 2011), samtools (Li et al., 2009), Bowtie 2 (Langmead & Salzberg, 2012), admixr (Petr n.d.), and custom python and bash scripts (see Github). To prepare for STRUCTURE and isolation by distance (IBD) analysis, we filtered snps using vcf tools (Danecek et al., 2011). We excluded snps with a minor allele count lower than 4 (equivalent to 2.2%) or with data missing in greater than 20% of samples. These thresholds were average to conservative based on those used in previous studies (Beck & Semple, 2015; Huang, Poland, Wight, Jackson, & Tinker, 2014; Ilut et al., 2015; Martin, Olsen, Samaniego, Zimmer, & Gilbert, 2016; McGrath, 2014; Mondon, Owens, Poverene, Cantamutto, & Rieseberg, 2017; Sawler et al., 2015; Taylor, Curry, White, Ferretti, & Lovette, 2014). We set the significance threshold for Hardy–Weinberg equilibrium at 1e‐5, which was the midpoint used in a review of past studies (Anderson et al., 2010). Since very rare alleles could still be important for assessing population structure (Linck & Battey, 2019), we evaluated whether inclusion of them altered the results and found that they did not. We present results with the exclusion of SNPs with rare minor allele counts < 2.2%.

### 2.6 Population structure and geography

To conduct a STRUCTURE analysis (Pritchard, Stephens, & Donnelly, 2000) while taking advantage of parallelization, we used StrAuto (Chhatre & Emerson, 2017).We conducted separate analyses for the two continents, with five replicates at K = 1–6 for each. In addition, we ran a STRUCTURE analysis using data from all the populations together. To visualize the STRUCTURE output, we used the default settings of the web‐based program Cluster Markov Packager Across K (CLUMPAK) (Kopelman, Mayzel, Jakobsson, Rosenberg, & Mayrose, 2015) and the R package *pophelper* (Francis, 2017).

To examine isolation by distance (IBD), we used the R package *adegenet* to test for IBD in each continent separately (Jombart, 2008). *Adegenet* uses a Mantel test between matrices of genetic and geographic distances to assess whether more spatially disparate populations are also more genetically divergent.

In addition to STRUCTURE and IBD, patterns in genetic data can also be understood with principal component analysis (Cavalli‐Sforza, Menozzi, & Piazza, 1994; Josephs, Berg, Ross‐Ibarra, & Coop, 2018; McVean, 2009; Patterson, Price, & Reich, 2006). We performed PCA on SNP data from the native and introduced ranges to test for correlations between major axes of SNP variation and the phenotypic traits of interest, and latitude. To extract principal components from SNPs, we used the R package LEA (Frichot & François, 2015). The vcf files were converted to geno files using the vcf2geno function. We then ran PCAs of all available SNPs separately for the two continents. To determine which principal components should be retained in subsequent analyses, we used Tracy–Widom tests (Patterson et al., 2006). For each population, we calculated a PC score along each significant axis of SNP variation and then used these PC scores to test for associations with all traits or geography. Specifically, we tested whether axes of neutral SNP variation were related to either geography or phenotype with linear regressions. For each continent, we regressed each significant principal component on latitude and the three phenotypic traits.

### 2.7 Descriptive population genetic statistics

To explore population genetics of the native and introduced ragweed populations, we used the programs Genodive and SplitsTree (Hudson, 1998; Meirmans & Van Tienderen, 2004). We converted vcf files for each continent, and for the entire dataset, to the genetix format and then imported them into Genodive. We then used Genodive to estimate F_ST_, population genetic summary statistics (including observed and expected heterozygosity and fixation indices), and to run an AMOVA to partition genetic variation between the range, population, and individual levels. We used SplitsTree to construct a NeighborNet for all the individuals (Hudson, 1998).

## 3 RESULTS

### 3.1 Phenotypic traits

**Figure 2:**
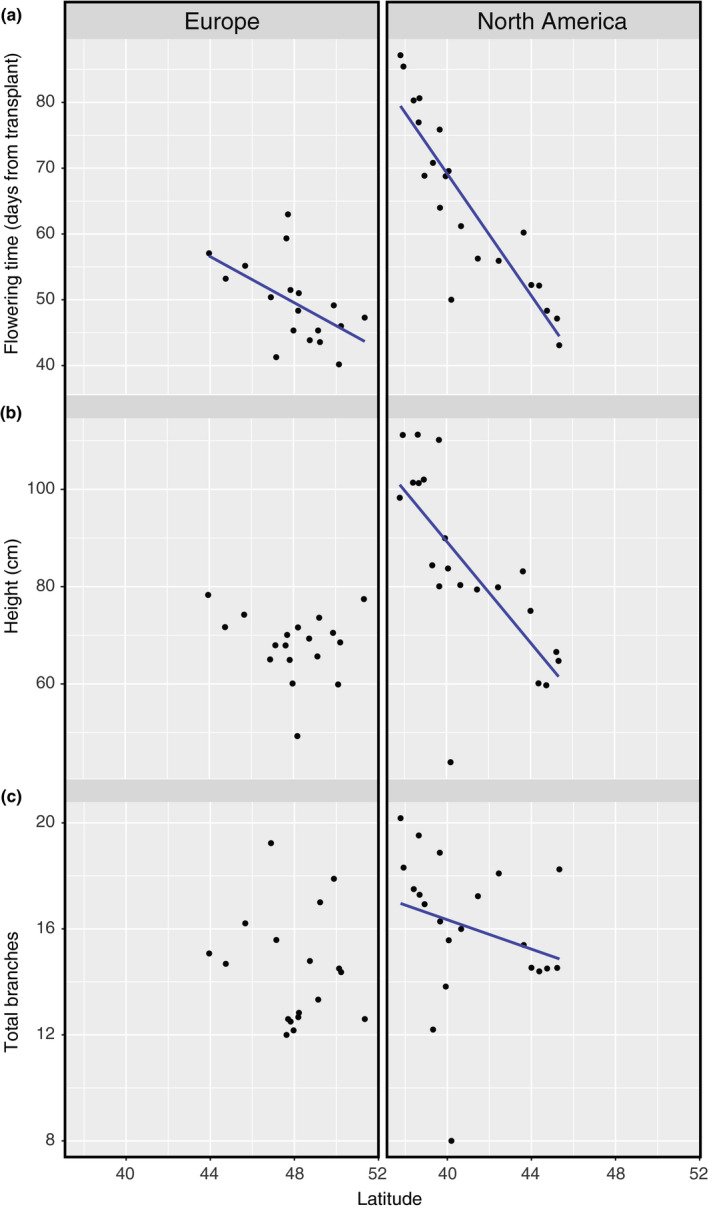
Phenotypic results for ragweed plants grown in common garden. (a) Flowering time, (b) height, and (c) total branches on the y‐axes and the latitudes of origin on the x‐axis

**Figure 3:**
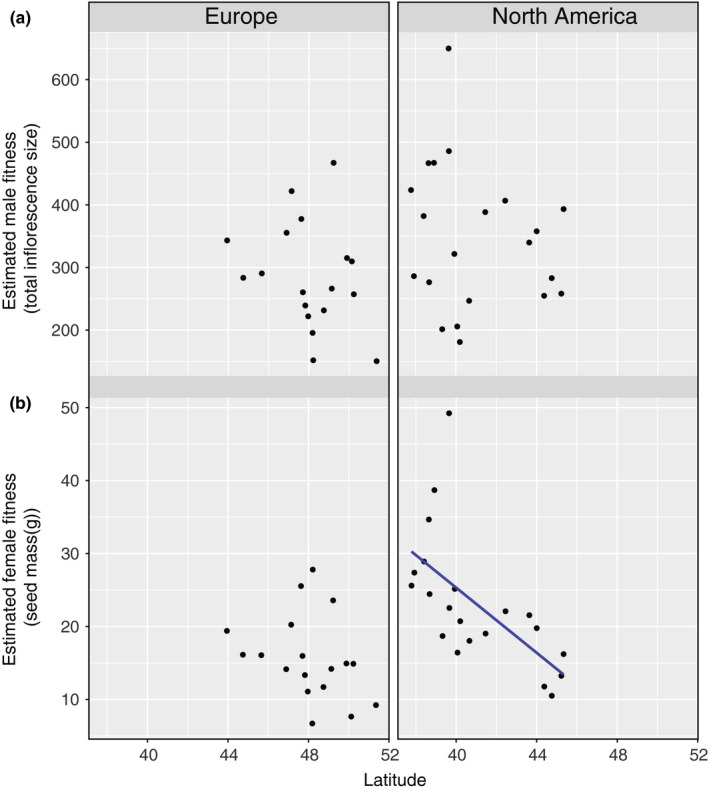
Female and male reproductive output results for ragweed plants grown in common garden. (a) Estimated male fitness and (b) estimated female fitness

Plants from more southern latitudes flowered later and grew larger both in total height and branch number (Figure2). These clines were all significant for the North American populations, while only flowering time had a statistically significant association with latitude in Europe (Table1). There was only one significant cline for fitness traits, with more southern North American plants producing more fruits than those in more northern populations (Figure3b). Since more southern plants were also larger, this correlation may be driven by a relationship between size and latitude.

**Table 1:** Result of regressions of *Ambrosia artemisiifolia* traits on latitude in the invasive (European) and native (North American) ranges

	Flowering time	Height	Total branches	Male fitness	Female fitness
EUROPE					
Estimate	−1.545***	−0.672	−0.120	−9.523	−0.578
Std. error	(0.319)	(0.578)	(0.180)	(7.236)	(0.387)
Degrees of freedom	231	228	227	228	194
NORTH AMERICA					
Estimate	−4.694***	−5.570***	−0.333***	−10.557	−2.243***
Std. error	(0.262)	(0.495)	(0.129)	(6.776)	(0.339)
Degrees of freedom	287	285	284	285	262

Regressions were performed on maternal family means.

*** indicates *p* < .01

### 3.2 Neutral markers

**Figure 4:**
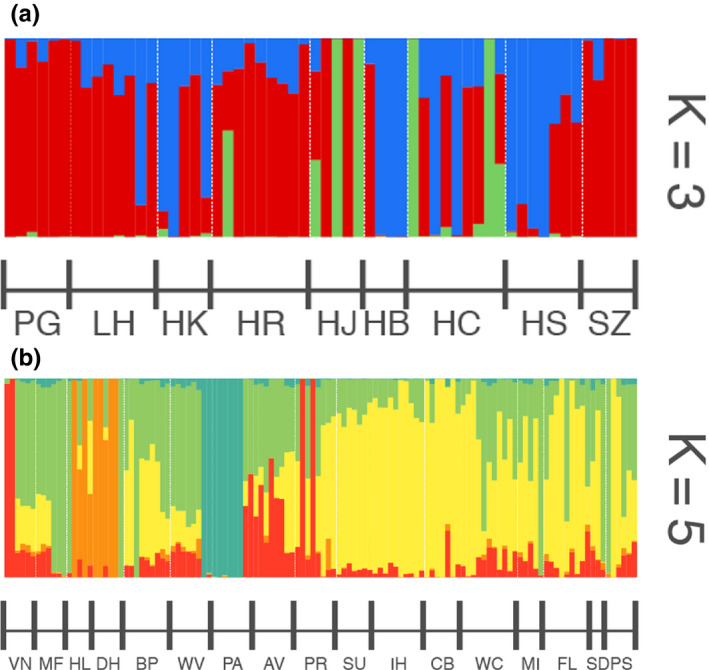
STRUCTURE results for ragweed populations (a) Europe and (b) North America. Populations are ordered from lowest to highest latitudes (south to north). Note that colors do not correspond between and (a) and (b)

**Figure A1:**
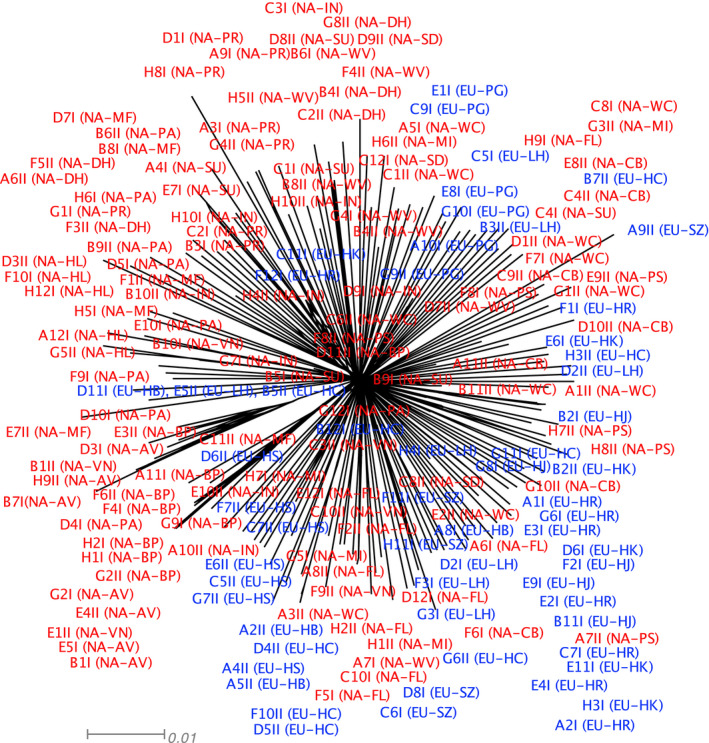
SplitsTree results for invasive and native ragweed populations

**Figure A2:**
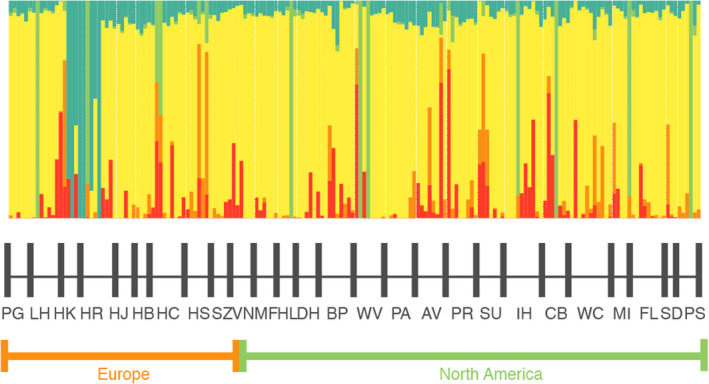
STRUCTURE results for all ragweed populations. Populations are ordered first by continent and then from lowest to highest latitudes within continents

The Illumina analysis resulted in 250 033 952 total sequences which all passed a FastQC quality check. We started with 258 540 SNPs across all the populations. After filtering, we had 20 843 sites for Europe and 28 643 for North America. Our STRUCTURE analysis indicated that the European samples clustered into three groups, while the North American lines clustered into five groups. In Europe, there was no obvious geographic pattern to ancestry (Figure4a). In North America, two populations seem distinct (dark green bars for population PA and light green bars for *SD*), but otherwise there is not much geographic structure (Figure4b). There was no significant isolation by distance in either continent (p‐value of 0.84 for Europe and 0.342 for North America). Our SplitsTree analysis was consistent with the above results, showing populations were not clustered into distinct subgroups (Figure A1). The global STRUCTURE analysis for all populations found the most support for k = 5. As with the by‐continent analyses, little of the ancestry was geographically structured (Figure A2).

Tracy–Widom tests indicated that there was one significant SNP principal component in Europe and seven for the North American dataset (North America: TW Statistic ≥ 1.029, *p* ≤ .04671 for PCs 1–7; Europe: TW Statistic = 6.255, *p* = 1.013E‐06 for PC1). The significant European principal component explained 4.5% of genetic variation. Altogether, the significant North America principal components explained 14.6% of genetic variation. There were no significant relationships between significant SNP principal component scores and either latitude or phenotype (all *p* values > .05). These results suggest that axes of (neutral) SNP variation are not related to either latitude or the phenotypic traits, suggesting that latitudinal clines are unlikely to be generated by neutral or stochastic processes.

In keeping with the lack of geographic structure revealed by the SplitsTree and STRUCTURE results, F_ST_ values were universally low (mean of 0.0639 [range of 0.003 to 0.213] for North America and 0.0566 [range of 0.018 to 0.12] for Europe; see Tables A3 & A4 for population‐specific results). Other population‐level statistics were consistent with the conclusion that there is low population differentiation in both ranges (Tables A5 & A6). When we used AMOVA on the global dataset, we detected very little variance at the between‐continent or between‐population levels (Table A7). Total heterozygosity was lower in native (0.150) as compared to the invasive range (0.202). The expected heterozygosity within populations was also higher in the invasive range (0.192 vs. 0.141 for the native range).

## 4 DISCUSSION

Introduced species offer a unique opportunity to address important questions in evolutionary biology (Sax et al., 2007; Yoshida et al. 2007). Adaptation is an important and sometimes overlooked aspect of invasions (Huey et al., 2005; Barrett 2015). New species introductions offer an excellent avenue to explore the rate and predictability of adaptation, a topic of great interest to evolutionary biologists (Huey et al., 2005). The reemergence of genetic clines in introduced ranges represents evidence that adaptation may occur quickly and result in predictable phenotypic differentiation (Leger & Rice, 2007). Adaptation to local habitats and population differentiation can be critical to the ability of an invasive species to expand its range (Colautti & Barrett, 2013). Here, we detected clinal differentiation in native and introduced populations of common ragweed and evidence that parallel clines in flowering time are the results of local adaptation as opposed to neutral processes. We also examined the population structure and genetic diversity of both native and invasive populations.

### 4.1 Clines as evidence of local adaptation

Past studies of ragweed have found geographic patterns in phenotype, including parallel clines in the native and various introduced ranges (van Boheeemen, Atwater, & Hodgins, 2019; Hodgins & Rieseberg, 2011; Li, She, Zhang, & Liao, 2015; Li, Zhang, et al., 2015). With populations from different parts of both Europe and North America than those used in previous studies, we corroborated their conclusions that flowering time patterns have been reproduced in the introduced range. We present evidence that these patterns are almost certainly the results of selection as opposed to neutral processes. Unlike flowering time, patterns of SNP variation were not correlated with latitude, and principal components of genetic variation were not correlated with the phenotypic traits we examined, including flowering time. Overall, there was little population structure and low F_ST_ values were identified in both ranges. Van Boheemen et al. (2019) detected repeated latitudinal divergence in a host of life history and size traits in Australian, North American, and European samples of ragweed, as well. In their case, they detected these clines when controlling for structure coefficients (so‐called q‐values) as a measure of neutral population genetic structure. Here, we used independent biological samples, phenotyped plants in a field common garden in the native range (as opposed to growth chamber), and used alternative means of assessing neutral population genetic structure (PCA of the SNP matrix). That we found qualitatively similar results—clinal differentiation above and beyond what could be explained by neutral processes—all make it unlikely that the cline in flowering time is the results of drift.

One caveat of this study is that all plants were grown from seed and therefore subject to maternal effects. However, we think it unlikely that maternal effects could explain the phenotypic patterns presented here. The population‐level maternal effects would all need to be in a consistent direction across latitude. Maternal effects are more pervasive in early‐life history stages (Montague et al., 2008; Rossiter, 1996), while here we focused on traits at the end of the annual life cycle. It is also unlikely that maternal effects would be responsible for differentiation at such large geographic scales (Montague et al., 2008). In addition, the clines found here were consistent with those of our previous work (McGoey & Stinchcombe, 2018), which included fewer populations but did remove the impact of maternal effects.

Ragweed has been present in Europe for ~250 years (Chauvel et al., 2006). In as many generations, it has spread and proliferated, especially in France, Italy, and the Pannonian Plain (Thibaudon, Šikoparija, Oliver, Smith, & Skjøth, 2014). Our previous research demonstrated that invasive ragweed populations possess ample additive genetic variation, in spite of any bottlenecks and founder effects during the colonization process. Given the results shown here, it must also be concluded that this quantitative genetic variation has been preserved in the face of selection and adaptation as well.

Past studies of population differentiation, including one examining ragweed in its introduced range, have used Q_ST_‐F_ST_ comparisons to assess geographic variation (Chun, Nason, & Moloney, 2009; Leinonen, O’hara, Cano, & Merilä, 2008). Chun and colleagues also found the invasive ragweed populations had low F_ST_ values and geographic structure (Chun et al., 2009), similar to our F_ST_ results. Their Q_ST_‐F_ST_ analysis indicated there was significant diversifying selection acting on ragweed in its introduced range. The conclusions of Chun and colleagues align with our own that population differentiation in the invasive range is the result of adaptive evolution as opposed to neutral processes. True Q_ST_ comparisons must obtain estimates of additive genetic variance, which means controlled crosses of dozens of pairs from each population. Doing so for six populations for previous work (McGoey & Stinchcombe, 2018) was challenging; controlled crosses to estimate additive genetic variance for more than thirty populations would be prohibitive.

### 4.2 Population genetics

Our population genetic results indicate that there is very little geographic population structure in both continents. Population differentiation in neutral markers as measured by F_ST_ was low for both the native (0.0639) and invasive (0.0566) ranges. Past work on ragweed has found similarly low F_ST_ values (van Boheemen et al., 2017; Chun, Fumanal, Laitung, & Bretagnolle, 2010; Chun, Corre, & Bretagnolle, 2011; Genton, Shykoff, & Giraud, 2005; Martin, 2011; Martin et al., 2016). Martin and colleagues used SNPs to assess population differentiation of North American ragweed and found that a solitary genetic cluster was the most likely population structure (Martin et al., 2016). Our STRUCTURE analyses showed the highest likelihoods for multiple clusters, but there was not a geographic pattern to the ancestral groupings, especially for the European populations. The consistent findings of low isolation by distance and population structure may not be surprising given that ragweed is a wind‐pollinated outcrosser (Friedman & Barrett, 2008).

The assumption that introduced species will always face significant deficits in genetic variation has been challenged by numerous counterexamples in the literature (Colautti & Lau, 2015; Estoup et al., 2016). In some cases, due to multiple introductions and subsequent admixture, molecular diversity is actually higher in invasive populations when compared to their native counterparts (Dlugosch & Parker, 2008; Keller & Taylor, 2008, 2010; Novak & Mack, 1993). Here, we estimated slightly higher metrics of genetic diversity (i.e., expected heterozygosity) in the invasive range for the represented populations. These findings corroborate a study in the French and North American ranges using microsatellite genetic variability where within‐population diversity was higher in the invasive range and overall genetic diversity was comparable between the two ranges (Genton et al., 2005). Similarly, Li, Liao, Wolfe, and Zhang (2012) found no reduction in genetic diversity between the native and introduced Chinese ranges using AFLP loci. The high genetic diversity in invasive ranges is likely the result of high propagule introduction from multiple sources in the native range (Genton et al. 2005a). The persistence of large, genetically diverse populations in the worst affected areas of Europe is dangerous source for the spread of ragweed into currently unaffected areas. Roads and railway tracks are ideal corridors and habitats for ragweed in the native range (Lavoie, Jodoin, & Merlis, 2007) and thus could also facilitate multiple introductions and gene flow in the invasive European range.

## 5 CONCLUSIONS

Adaptation in introduced environments is not just theoretically interesting, but also has extremely important ecological and economic implications. Gradients in abiotic variables can lead to divergent selection across an introduced range and, if populations have sufficient genetic variation, create clines in traits (Maron, Vilà, Bommarco, Elmendorf, & Beardsley, 2004). This adaptation can exacerbate the negative impacts of introduced species (Huey et al., 2005; Maron et al., 2004).

Ragweed has already caused economic and health impacts in Europe (Buttenschøn et al., 2010). Climate change is expected to extend its growing season, and it continues to spread its geographic range (Ziska et al. 2011). Our research corroborates past findings that ragweed has been able to adapt to its invasive range (Chun et al., 2011; Hodgins & Rieseberg, 2011). The genetic, phenotypic, and ecological traits of introduced ragweed make it very likely that the invasion will worsen.

## AUTHOR CONTRIBUTIONS

**Brechann V. McGoey:** Conceptualization (equal); Data curation (equal); Formal analysis (equal); Investigation (equal); Methodology (equal); Project administration (equal); Writing‐original draft (equal); Writing‐review & editing (equal). **Kathryn A. Hodgins:** Conceptualization (equal); Investigation (equal); Methodology (equal); Project administration (equal); Resources (equal); Writing‐review & editing (equal). **John R. Stinchcombe:** Conceptualization (equal); Funding acquisition (equal); Project administration (equal); Resources (equal); Supervision (equal); Writing‐original draft (equal); Writing‐review & editing (equal). 

BVM and JRS designed the study; BVM and KAH carried out the study; BVM and KAH analyzed the data; BVM wrote the manuscript; and BVM, KAH, and JRS edited, commented, and revised the manuscript.

## Data Availability

Sequences deposited in the short read archive are available from bioproject accession #PRJNA607570 (https://www.ncbi.nlm.nih.gov/sra). Phenotypic data have been deposited in Dryad (https://doi.org/10.5061/dryad.d51c59zzq). A summary of our bioinformatics pipeline, including code and links to the original fastq files, is available here (https://github.com/brechann‐mcgoey/ragweedGBS).

## Appendices 1

**Table A1:** Population coordinates for 18 European (invasive) ragweed populations

Population Abbreviation	Continent	Latitude	Longitude
PG	Europe	43.9475	4.536631
RM	Europe	44.74194	4.940783
LH	Europe	45.66117	4.966539
HK	Europe	46.89164819	19.65085249
HR	Europe	47.13845	19.47464
HM	Europe	47.62666192	19.44151603
HJ	Europe	47.70273145	20.07493272
HB	Europe	47.8226962	19.089972
HC	Europe	47.962356	20.894481
HS	Europe	48.18515189	20.92252985
SH	Europe	48.21292953	18.93131109
SZ	Europe	48.74195476	19.13689272
SB	Europe	49.13566482	21.55096851
SL	Europe	49.222985	19.302752
PV	Europe	49.8798343	19.89245121
PB	Europe	50.12194	18.81494
PH	Europe	50.22392429	19.17026453
PE	Europe	51.35332297	19.60609006

**Table A2:** Population coordinates for 20 North American (native) ragweed populations

Population Abbreviation	Continent	Latitude	Longitude
OM	North American	37.77932728	−75.61065442
VN	North American	37.92026101	−75.47660198
MF	North American	38.40708263	−75.56824697
DS	North American	38.64183334	−75.445
SC	North American	38.68109706	−75.07450333
HL	North American	38.92013797	−75.44832488
DH	North American	39.32390625	−75.62242479
BP	North American	39.64569554	−75.71813984
WV	North American	39.6584	−79.90525
PA	North American	39.930872	−75.583156
AV	North American	40.20018743	−76.76244026
PR	North American	40.65946579	−76.91820217
SU	North American	41.45751046	−77.13565654
IH	North American	42.44996	−76.46113
CB	North American	43.63519	−79.34576
WC	North American	44.003197	−79.393311
MI	North American	44.37683	−79.74113
FL	North American	44.750014	−79.704694
*SD*	North American	45.225364	−79.681836
PS	North American	45.3347	−79.956231

**Table A3:** Pairwise F_st_ values for North American ragweed populations

	VN	MF	HL	BP	WV	PA	AV	PR	SU	CB	MI	FL	*SD*	PS	DH	IH	WC
VN	‐‐	0.061	0.101	0.18	0.078	0.082	0.18	0.089	0.093	0.075	0.073	0.061	0.13	0.085	0.086	0.066	0.063
MF	0.061	‐‐	0.033	0.118	0.02	0.018	0.116	0.031	0.029	0.017	0.018	0.022	0.049	0.027	0.02	0.007	0.015
HL	0.101	0.033	‐‐	0.154	0.051	0.052	0.149	0.059	0.061	0.049	0.05	0.054	0.092	0.057	0.053	0.04	0.047
BP	0.18	0.118	0.154	‐‐	0.131	0.131	0.213	0.137	0.145	0.129	0.139	0.13	0.188	0.145	0.139	0.117	0.122
WV	0.078	0.02	0.051	0.131	‐‐	0.034	0.125	0.044	0.038	0.024	0.019	0.026	0.049	0.03	0.043	0.02	0.02
PA	0.082	0.018	0.052	0.131	0.034	‐‐	0.125	0.043	0.043	0.033	0.032	0.035	0.068	0.044	0.042	0.021	0.029
AV	0.18	0.116	0.149	0.213	0.125	0.125	‐‐	0.133	0.135	0.124	0.129	0.121	0.171	0.137	0.136	0.112	0.114
PR	0.089	0.031	0.059	0.137	0.044	0.043	0.133	‐‐	0.051	0.04	0.039	0.042	0.072	0.049	0.053	0.031	0.036
SU	0.093	0.029	0.061	0.145	0.038	0.043	0.135	0.051	‐‐	0.035	0.036	0.036	0.079	0.044	0.05	0.025	0.031
CB	0.075	0.017	0.049	0.129	0.024	0.033	0.124	0.04	0.035	‐‐	0.009	0.009	0.046	0.02	0.043	0.015	0.007
MI	0.073	0.018	0.05	0.139	0.019	0.032	0.129	0.039	0.036	0.009	‐‐	0.01	0.041	0.011	0.042	0.016	0.003
FL	0.061	0.022	0.054	0.13	0.026	0.035	0.121	0.042	0.036	0.009	0.01	‐‐	0.04	0.017	0.041	0.017	0.006
*SD*	0.13	0.049	0.092	0.188	0.049	0.068	0.171	0.072	0.079	0.046	0.041	0.04	‐‐	0.054	0.071	0.047	0.036
PS	0.085	0.027	0.057	0.145	0.03	0.044	0.137	0.049	0.044	0.02	0.011	0.017	0.054	‐‐	0.048	0.025	0.012
DH	0.086	0.02	0.053	0.139	0.043	0.042	0.136	0.053	0.05	0.043	0.042	0.041	0.071	0.048	‐‐	0.031	0.037
IH	0.066	0.007	0.04	0.117	0.02	0.021	0.112	0.031	0.025	0.015	0.016	0.017	0.047	0.025	0.031	‐‐	0.009
WC	0.063	0.015	0.047	0.122	0.02	0.029	0.114	0.036	0.031	0.007	0.003	0.006	0.036	0.012	0.037	0.009	‐‐

**Table A4:** Pairwise F_st_ values for European ragweed populations

	PG	LH	HK	HR	HJ	HB	HC	HS	SZ
PG	‐‐	0.021	0.050	0.026	0.091	0.051	0.043	0.038	0.054
LH	0.021	‐‐	0.035	0.018	0.083	0.034	0.035	0.030	0.047
HK	0.050	0.035	‐‐	0.036	0.105	0.063	0.056	0.050	0.07
HR	0.026	0.018	0.036	‐‐	0.080	0.037	0.039	0.028	0.046
HJ	0.091	0.083	0.105	0.080	‐‐	0.120	0.099	0.092	0.114
HB	0.051	0.034	0.063	0.037	0.120	‐‐	0.061	0.051	0.071
HC	0.043	0.035	0.056	0.039	0.099	0.061	‐‐	0.046	0.063
HS	0.038	0.030	0.050	0.028	0.092	0.051	0.046	‐‐	0.058
SZ	0.054	0.047	0.070	0.046	0.114	0.071	0.063	0.058	‐‐

**Table A5:** Population genetic summary statistics for North American ragweed

Statistic	Value	Std.Dev.	c.i.2.5%	c.i.97.5%	Description
Num	2	0	2	2	Number of alleles
Eff_num	1.163	0.001	1.161	1.165	Effective number of alleles
Ho	0.108	0.001	0.107	0.11	Observed heterozygosity
Hs	0.141	0.001	0.14	0.142	Heterozygosity within populations
Ht	0.15	0.001	0.148	0.151	Total heterozygosity
H't	0.15	0.001	0.149	0.152	Corrected total heterozygosity
Gis	0.23	0.001	0.228	0.233	Inbreeding coefficient
Gst	0.061	0.001	0.06	0.062	Fixation index
G'st(Nei)	0.064	0.001	0.063	0.065	Nei, corrected fixation index
G'st(Hed)	0.071	0.001	0.07	0.072	Hedrick, standardised fixation index
G''st	0.075	0.001	0.073	0.076	Corrected standardised fixation index
D_est	0.011	0	0.011	0.011	Jost, differentiation

**Table A6:** Population genetic summary statistics for European ragweed

Statistic	Value	Std.Dev.	c.i.2.5%	c.i.97.5%	Description
Num	2	0	2	2	Number of alleles
Eff_num	1.224	0.001	1.221	1.226	Effective number of alleles
Ho	0.137	0.001	0.136	0.138	Observed heterozygosity
Hs	0.192	0.001	0.19	0.193	Heterozygosity within populations
Ht	0.202	0.001	0.2	0.204	Total heterozygosity
H't	0.203	0.001	0.201	0.205	Corrected total heterozygosity
Gis	0.285	0.002	0.281	0.289	Inbreeding coefficient
Gst	0.051	0.001	0.05	0.052	Fixation index
G'st(Nei)	0.057	0.001	0.055	0.059	Nei, corrected fixation index
G'st(Hed)	0.065	0.001	0.063	0.066	Hedrick, standardised fixation index
G''st	0.071	0.001	0.069	0.073	Corrected standardised fixation index
D_est	0.014	0	0.014	0.015	Jost, differentiation

**Table A7:** AMOVA output for North American and European ragweed

Source of Variation	Nested in	%var	F‐stat	*F*‐value	Std.Dev.	c.i.2.5%	c.i.97.5%	P‐value	F'‐value
Within Individual	‐‐	0.795	F_it_	0.205	0.001	0.204	0.207	‐‐	‐‐
Among Individual	Population	0.162	F_is_	0.169	0.001	0.167	0.171	0.001	‐‐
Among Population	Continent	0.042	F_sc_	0.042	0	0.041	0.043	0.001	0.048
Among Continents	‐‐	0.002	F_ct_	0.002	0	0.001	0.002	0.017	0.002
