# Supplementary figures and images for: Parallel flowering time clines in native and introduced ragweed populations are likely due to adaptation
Source: Ecol Evol. 2020 Apr 29;10(11):4595–608. doi: 10.1002/ece3.6163 (PMC7297792; doi:10.1002/ece3.6163)

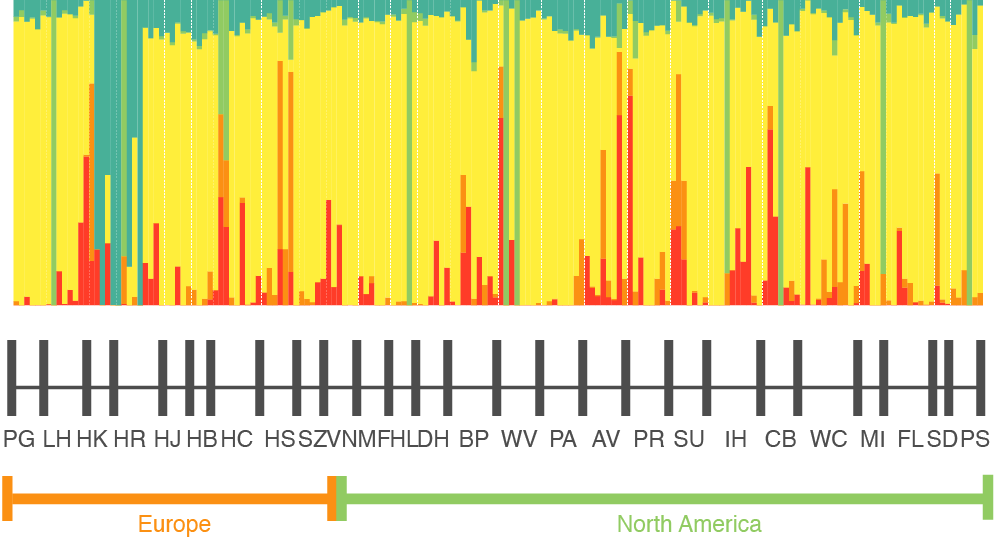

Supplement: Supplementary file 2 [file ECE3-10-4595-s002.png]
