# Supplementary material for: Parallel flowering time clines in native and introduced ragweed populations are likely due to adaptation
Source: Ecol Evol. 2020 Apr 29;10(11):4595–608. doi: 10.1002/ece3.6163 (PMC7297792; doi:10.1002/ece3.6163)
Supplement: Supplementary file 3 [file ECE3-10-4595-s003.docx]

**Appendices**

**Table A1.** Population coordinates for 18 European (invasive) ragweed populations.

| **Population Abbreviation** | **Continent** | **Latitude** | **Longitude** |
| --- | --- | --- | --- |
| PG | Europe | 43.9475 | 4.536631 |
| RM | Europe | 44.74194 | 4.940783 |
| LH | Europe | 45.66117 | 4.966539 |
| HK | Europe | 46.89164819 | 19.65085249 |
| HR | Europe | 47.13845 | 19.47464 |
| HM | Europe | 47.62666192 | 19.44151603 |
| HJ | Europe | 47.70273145 | 20.07493272 |
| HB | Europe | 47.8226962 | 19.089972 |
| HC | Europe | 47.962356 | 20.894481 |
| HS | Europe | 48.18515189 | 20.92252985 |
| SH | Europe | 48.21292953 | 18.93131109 |
| SZ | Europe | 48.74195476 | 19.13689272 |
| SB | Europe | 49.13566482 | 21.55096851 |
| SL | Europe | 49.222985 | 19.302752 |
| PV | Europe | 49.8798343 | 19.89245121 |
| PB | Europe | 50.12194 | 18.81494 |
| PH | Europe | 50.22392429 | 19.17026453 |
| PE | Europe | 51.35332297 | 19.60609006 |

**Table A2.** Population coordinates for 20 North American (native) ragweed populations.

| **Population Abbreviation** | **Continent** | **Latitude** | **Longitude** |
| --- | --- | --- | --- |
| OM | North American | 37.77932728 | -75.61065442 |
| VN | North American | 37.92026101 | -75.47660198 |
| MF | North American | 38.40708263 | -75.56824697 |
| DS | North American | 38.64183334 | -75.445 |
| SC | North American | 38.68109706 | -75.07450333 |
| HL | North American | 38.92013797 | -75.44832488 |
| DH | North American | 39.32390625 | -75.62242479 |
| BP | North American | 39.64569554 | -75.71813984 |
| WV | North American | 39.6584 | -79.90525 |
| PA | North American | 39.930872 | -75.583156 |
| AV | North American | 40.20018743 | -76.76244026 |
| PR | North American | 40.65946579 | -76.91820217 |
| SU | North American | 41.45751046 | -77.13565654 |
| IH | North American | 42.44996 | -76.46113 |
| CB | North American | 43.63519 | -79.34576 |
| WC | North American | 44.003197 | -79.393311 |
| MI | North American | 44.37683 | -79.74113 |
| FL | North American | 44.750014 | -79.704694 |
| SD | North American | 45.225364 | -79.681836 |
| PS | North American | 45.3347 | -79.956231 |

**Table A3**. Pairwise F_st_ values for North American ragweed populations

|  | VN | MF | HL | BP | WV | PA | AV | PR | SU | CB | MI | FL | SD | PS | DH | IH | WC |
| --- | --- | --- | --- | --- | --- | --- | --- | --- | --- | --- | --- | --- | --- | --- | --- | --- | --- |
| VN | -- | 0.061 | 0.101 | 0.18 | 0.078 | 0.082 | 0.18 | 0.089 | 0.093 | 0.075 | 0.073 | 0.061 | 0.13 | 0.085 | 0.086 | 0.066 | 0.063 |
| MF | 0.061 | -- | 0.033 | 0.118 | 0.02 | 0.018 | 0.116 | 0.031 | 0.029 | 0.017 | 0.018 | 0.022 | 0.049 | 0.027 | 0.02 | 0.007 | 0.015 |
| HL | 0.101 | 0.033 | -- | 0.154 | 0.051 | 0.052 | 0.149 | 0.059 | 0.061 | 0.049 | 0.05 | 0.054 | 0.092 | 0.057 | 0.053 | 0.04 | 0.047 |
| BP | 0.18 | 0.118 | 0.154 | -- | 0.131 | 0.131 | 0.213 | 0.137 | 0.145 | 0.129 | 0.139 | 0.13 | 0.188 | 0.145 | 0.139 | 0.117 | 0.122 |
| WV | 0.078 | 0.02 | 0.051 | 0.131 | -- | 0.034 | 0.125 | 0.044 | 0.038 | 0.024 | 0.019 | 0.026 | 0.049 | 0.03 | 0.043 | 0.02 | 0.02 |
| PA | 0.082 | 0.018 | 0.052 | 0.131 | 0.034 | -- | 0.125 | 0.043 | 0.043 | 0.033 | 0.032 | 0.035 | 0.068 | 0.044 | 0.042 | 0.021 | 0.029 |
| AV | 0.18 | 0.116 | 0.149 | 0.213 | 0.125 | 0.125 | -- | 0.133 | 0.135 | 0.124 | 0.129 | 0.121 | 0.171 | 0.137 | 0.136 | 0.112 | 0.114 |
| PR | 0.089 | 0.031 | 0.059 | 0.137 | 0.044 | 0.043 | 0.133 | -- | 0.051 | 0.04 | 0.039 | 0.042 | 0.072 | 0.049 | 0.053 | 0.031 | 0.036 |
| SU | 0.093 | 0.029 | 0.061 | 0.145 | 0.038 | 0.043 | 0.135 | 0.051 | -- | 0.035 | 0.036 | 0.036 | 0.079 | 0.044 | 0.05 | 0.025 | 0.031 |
| CB | 0.075 | 0.017 | 0.049 | 0.129 | 0.024 | 0.033 | 0.124 | 0.04 | 0.035 | -- | 0.009 | 0.009 | 0.046 | 0.02 | 0.043 | 0.015 | 0.007 |
| MI | 0.073 | 0.018 | 0.05 | 0.139 | 0.019 | 0.032 | 0.129 | 0.039 | 0.036 | 0.009 | -- | 0.01 | 0.041 | 0.011 | 0.042 | 0.016 | 0.003 |
| FL | 0.061 | 0.022 | 0.054 | 0.13 | 0.026 | 0.035 | 0.121 | 0.042 | 0.036 | 0.009 | 0.01 | -- | 0.04 | 0.017 | 0.041 | 0.017 | 0.006 |
| SD | 0.13 | 0.049 | 0.092 | 0.188 | 0.049 | 0.068 | 0.171 | 0.072 | 0.079 | 0.046 | 0.041 | 0.04 | -- | 0.054 | 0.071 | 0.047 | 0.036 |
| PS | 0.085 | 0.027 | 0.057 | 0.145 | 0.03 | 0.044 | 0.137 | 0.049 | 0.044 | 0.02 | 0.011 | 0.017 | 0.054 | -- | 0.048 | 0.025 | 0.012 |
| DH | 0.086 | 0.02 | 0.053 | 0.139 | 0.043 | 0.042 | 0.136 | 0.053 | 0.05 | 0.043 | 0.042 | 0.041 | 0.071 | 0.048 | -- | 0.031 | 0.037 |
| IH | 0.066 | 0.007 | 0.04 | 0.117 | 0.02 | 0.021 | 0.112 | 0.031 | 0.025 | 0.015 | 0.016 | 0.017 | 0.047 | 0.025 | 0.031 | -- | 0.009 |
| WC | 0.063 | 0.015 | 0.047 | 0.122 | 0.02 | 0.029 | 0.114 | 0.036 | 0.031 | 0.007 | 0.003 | 0.006 | 0.036 | 0.012 | 0.037 | 0.009 | -- |

**Table A4**. Pairwise F_st_ values for European ragweed populations

|  | PG | LH | HK | HR | HJ | HB | HC | HS | SZ |
| --- | --- | --- | --- | --- | --- | --- | --- | --- | --- |
| PG | -- | 0.021 | 0.050 | 0.026 | 0.091 | 0.051 | 0.043 | 0.038 | 0.054 |
| LH | 0.021 | -- | 0.035 | 0.018 | 0.083 | 0.034 | 0.035 | 0.030 | 0.047 |
| HK | 0.050 | 0.035 | -- | 0.036 | 0.105 | 0.063 | 0.056 | 0.050 | 0.07 |
| HR | 0.026 | 0.018 | 0.036 | -- | 0.080 | 0.037 | 0.039 | 0.028 | 0.046 |
| HJ | 0.091 | 0.083 | 0.105 | 0.080 | -- | 0.120 | 0.099 | 0.092 | 0.114 |
| HB | 0.051 | 0.034 | 0.063 | 0.037 | 0.120 | -- | 0.061 | 0.051 | 0.071 |
| HC | 0.043 | 0.035 | 0.056 | 0.039 | 0.099 | 0.061 | -- | 0.046 | 0.063 |
| HS | 0.038 | 0.030 | 0.050 | 0.028 | 0.092 | 0.051 | 0.046 | -- | 0.058 |
| SZ | 0.054 | 0.047 | 0.070 | 0.046 | 0.114 | 0.071 | 0.063 | 0.058 | -- |

**Table A5**. Population genetic summary statistics for North American ragweed

| Statistic | Value | Std.Dev. | c.i.2.5% | c.i.97.5% | Description |
| --- | --- | --- | --- | --- | --- |
| Num | 2 | 0 | 2 | 2 | Number of alleles |
| Eff_num | 1.163 | 0.001 | 1.161 | 1.165 | Effective number of alleles |
| Ho | 0.108 | 0.001 | 0.107 | 0.11 | Observed heterozygosity |
| Hs | 0.141 | 0.001 | 0.14 | 0.142 | Heterozygosity within populations |
| Ht | 0.15 | 0.001 | 0.148 | 0.151 | Total heterozygosity |
| H't | 0.15 | 0.001 | 0.149 | 0.152 | Corrected total heterozygosity |
| Gis | 0.23 | 0.001 | 0.228 | 0.233 | Inbreeding coefficient |
| Gst | 0.061 | 0.001 | 0.06 | 0.062 | Fixation index |
| G'st(Nei) | 0.064 | 0.001 | 0.063 | 0.065 | Nei, corrected fixation index |
| G'st(Hed) | 0.071 | 0.001 | 0.07 | 0.072 | Hedrick, standardised fixation index |
| G''st | 0.075 | 0.001 | 0.073 | 0.076 | Corrected standardised fixation index |
| D_est | 0.011 | 0 | 0.011 | 0.011 | Jost, differentiation |

**Table A6**. Population genetic summary statistics for European ragweed

| Statistic | Value | Std.Dev. | c.i.2.5% | c.i.97.5% | Description |
| --- | --- | --- | --- | --- | --- |
| Num | 2 | 0 | 2 | 2 | Number of alleles |
| Eff_num | 1.224 | 0.001 | 1.221 | 1.226 | Effective number of alleles |
| Ho | 0.137 | 0.001 | 0.136 | 0.138 | Observed heterozygosity |
| Hs | 0.192 | 0.001 | 0.19 | 0.193 | Heterozygosity within populations |
| Ht | 0.202 | 0.001 | 0.2 | 0.204 | Total heterozygosity |
| H't | 0.203 | 0.001 | 0.201 | 0.205 | Corrected total heterozygosity |
| Gis | 0.285 | 0.002 | 0.281 | 0.289 | Inbreeding coefficient |
| Gst | 0.051 | 0.001 | 0.05 | 0.052 | Fixation index |
| G'st(Nei) | 0.057 | 0.001 | 0.055 | 0.059 | Nei, corrected fixation index |
| G'st(Hed) | 0.065 | 0.001 | 0.063 | 0.066 | Hedrick, standardised fixation index |
| G''st | 0.071 | 0.001 | 0.069 | 0.073 | Corrected standardised fixation index |
| D_est | 0.014 | 0 | 0.014 | 0.015 | Jost, differentiation |

**Table A7**. AMOVA output for North American and European ragweed

| Source of Variation | Nested in | %var | F-stat | F-value | Std.Dev. | c.i.2.5% | c.i.97.5% | P-value | F'-value |
| --- | --- | --- | --- | --- | --- | --- | --- | --- | --- |
| Within Individual | -- | 0.795 | F_it_ | 0.205 | 0.001 | 0.204 | 0.207 | -- | -- |
| Among Individual | Population | 0.162 | F_is_ | 0.169 | 0.001 | 0.167 | 0.171 | 0.001 | -- |
| Among Population | Continent | 0.042 | F_sc_ | 0.042 | 0 | 0.041 | 0.043 | 0.001 | 0.048 |
| Among Continents | -- | 0.002 | F_ct_ | 0.002 | 0 | 0.001 | 0.002 | 0.017 | 0.002 |
